# *Drosophila simulans* – *Ilp2*

**DOI:** 10.17912/micropub.biology.000679

**Published:** 2022-11-15

**Authors:** Leon F. Laskowski, Cole A. Kiser, Robyn Huber, Sean Kusche, Andrew M Arsham, Thomas C. Giarla, Chinmay P. Rele

**Affiliations:** 1 University of St. Francis, Joliet, IL, USA; 2 University of Alabama, Tuscaloosa, AL, USA; 3 Bemidji State University, Bemidji, MN, USA; 4 Siena College, Loudonville, NY, USA

**Figure 1.**
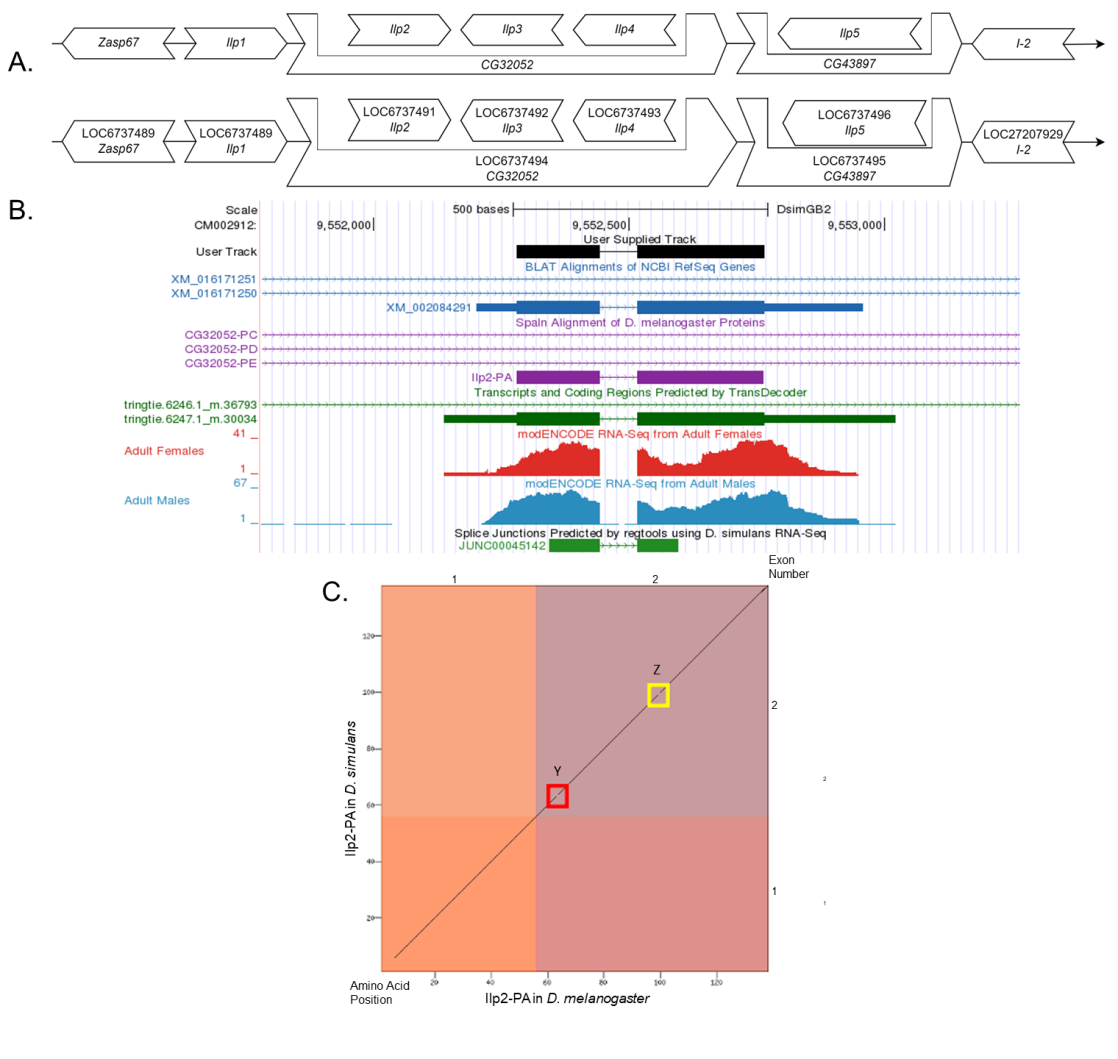
**
(A) Synteny comparison of the genomic neighborhoods for *Ilp2* in *Drosophila melanogaster* and *D. simulans* .** Thin underlying arrows indicate the DNA strand within which the target gene– *Ilp2* –is located in *D. melanogaster* (top) and *D. simulans* (bottom). Thin arrow(s) pointing to the right indicate(s) that *Ilp2* is on the positive (+) strand in *D. simulans* and *D.melanogaster* . The wide gene arrows pointing in the same direction as *Ilp2* are on the same strand relative to the thin underlying arrows, while wide gene arrows pointing in the opposite direction of *Ilp2* are on the opposite strand relative to the thin underlying arrows. White gene arrows in *D. simulans* indicate orthology to the corresponding gene in *D. melanogaster* . Gene symbols given in the *D. simulans* gene arrows indicate the orthologous gene in *D. melanogaster* , while the locus identifiers are specific to *D. simulans* . **(B) Gene Model in GEP UCSC Track Data Hub** (Raney *et al* ., 2014). The coding-regions of *Ilp2* in *D. simulans* are displayed in the User Supplied Track (black); coding exons are depicted by thick rectangles and introns by thin lines with arrows indicating the direction of transcription. Subsequent evidence tracks include BLAT Alignments of NCBI RefSeq Genes (dark blue, alignment of Ref-Seq genes for *D. simulans* ), Spaln of D. melanogaster Proteins (purple, alignment of Ref-Seq proteins from *D. melanogaster* ), Transcripts and Coding Regions Predicted by TransDecoder (dark green), RNA-Seq from Adult Females and Adult Males (red and light blue, respectively; alignment of Illumina RNA-Seq reads from *D. simulans* ), and Splice Junctions Predicted by regtools using *D. simulans* RNA-Seq (Graveley *et al.* , 2011; SRP006203). Splice junctions shown have a read-depth of 50-99 supporting reads in green. **(C) Dot Plot of Ilp2-PA in *D. melanogaster* ( *x* -axis) vs. the orthologous peptide in *D. simulans* ( *y* -axis).** Amino acid number is indicated along the left and bottom; coding-exon number is indicated along the top and right, and exons are also highlighted with alternating colors. The boxes highlighted in red and yellow (Box Y; Box Z) indicate lack of sequence similarity between amino acids.

## Description

***Introduction*** The Insulin Signaling Pathway is a highly conserved signaling pathway in animals, and is central to nutrient uptake (Hietakangas and Cohen 2009, Grewal 2009). *Ilp2* (aka *dilp2, Dilp 2, DILP, dilp-2, Ilp-2* ), a member of the Insulin Signaling Pathway, mediates growth by acting as a ligand for the insulin receptor and transducing a signal via the Chico/PI3K/Akt(PKB) pathway (Brogiolo *et al.* , 2001, Park *et al.* , 2014). *Ilp2* plays a role in regulating body size by increasing cell size and cell number of individual organs (Ren *et al.* , 2017). Evolutionary studies showed that loss of *Ilp2* increased lifespan, and changes in *Ilp2* expression may have contributed to the evolution of body size in the Hawaiian *Drosophila* species (Grönke *et al.* , 2010). In the absence of *Ilp2* , over-expression of *Ilp1* and *Ilp3-7* is enough to promote growth in *Drosophila* (Ikeya *et al.* , 2002). *Ilp2* mutants also seem to have severe developmental delay (Grönke *et al.* , 2010).

We propose a gene model for the *D. simulans* ortholog of the *D. melanogaster* Insulin-like peptide 2 *(Ilp2)* gene. The genomic region of the ortholog corresponds to the uncharacterized protein LOC6737491 (RefSeq accession XP_002084327.1) in the ASM75419v3 Genome Assembly of *D. simulans* (GenBank Accession: GCA_000754195.3 - Graveley *et al.* , 2011; SRP006203). This model is based on RNA-Seq data from *D. simulans* (Graveley *et al.* , 2011; SRP006203) and *Ilp2* in *D. melanogaster* using FlyBase release FB2022_04 (GCA_000001215.4; Larkin *et al.* , 2021). *D. simulans* is part of the melanogaster species group within the subgenus *Sophophora* of the genus *Drosophila* (Sturtevant, 1939; Bock and Wheeler, 1972). It was first described by Sturtevant (1919). *D. simulans* is a sibling species to *D. melanogaster* , thus extensively studied in the context of speciation genetics and evolutionary ecology (Powell, 1990). Historically, *D. simulans* was a tropical species native to sub-Saharan Africa (Lemeunier *et al* ., 1986) where figs served as a primary host (Lachaise and Tsacas, 1983). However, *D. simulans* ’s range has expanded world-wide within the last century as a human commensal using a broad range of rotting fruits as breeding sites (https://www.taxodros.uzh.ch). The Genomics Education Partnership maintains a mirror of the UCSC Genome Browser (Kent WJ *et al.* , 2002; Gonzalez *et al.* , 2021), which is available at https://gander.wustl.edu .

***Synteny***

The target gene, *Ilp2,* occurs on chromosome 3L in *D. melanogaster* and is flanked upstream by Z band alternatively spliced PDZ-motif protein 67 *(Zasp67),* Insulin-like peptide *(Ilp1)* and *CG32052,* which nests *Ilp2, Ilp3* and *Ilp4* . *Ilp2* is flanked downstream by Insulin-like peptide 3 *(Ilp3)* , Insulin-like peptide 4 *(Ilp4),* Insulin-like peptide 5 *(Ilp5)* (nested within *CG43897* ), and Inhibitor-2 *(I-2)* . The *tblastn* search of *D. melanogaster* Ilp2-PA (query) against the *D. simulans* (GenBank Accession: GCA_000754195.3) Genome Assembly (database) placed the putative ortholog of *Ilp2* within scaffold chromosome 3L (CM002912.1) at locus LOC6737491 (XP_002084327.1)— with an E-value of 5e-97 and a percent identity of 97.81%. Furthermore, the putative ortholog is flanked upstream by LOC6737489 (XP_016031285.1), LOC6737490 (XP_002084326.1) and LOC6737494 (XP_016031282.1), which correspond to *Zasp67* , *Ilp1* and *CG32052* in *D. melanogaster* (E-value: 0.0, 3e-78 and 0.0; identity: 95.60%, 91.67% and 98.62%, respectively, as determined by *blastp* ; Figure 1A, Altschul *et al.* , 1990). The putative ortholog of *Ilp2* is flanked downstream by LOC6737492 (XP_002084328.1), LOC6737493 (XP_002084329.1), LOC6737496 (XP_002084332.2) nested by LOC6737495 (XP_039149023.1), and LOC27207929 (XP_016031291.1), which correspond to *Ilp3* , *Ilp4, Ilp5, CG43897,* and *I-2* in *D. melanogaster* (E-value: 4e-59, 2e-58, 5e-52, 0.0, and 1e-128; identity: 98.33%, 97.35%, 97.22%, 92.64%, and 91.32% respectively, as determined by *blastp* ). The putative ortholog assignment for *Ilp2* in *D. simulans* is supported by the following evidence: The genes surrounding the *Ilp2* ortholog are orthologous to the genes at the same locus in *D. melanogaster* and local synteny is completely conserved, supported by e-values and percent identities, so we conclude that LOC6737491 is the correct ortholog of *Ilp2* in *D. simulans* (Figure 1A).

***Protein Model***

Consistent with the *blastp* search result which shows 97.81% identity between *D. melanogaster* Ilp2-PA and the *D. simulans* gene model as well as the low sensitivity parameters used to generate the dot plot (i.e., word size = 3; neighborhood threshold = 11), the dot plot of the two protein sequences contain multiple short gaps along the diagonal (Figure 1C).The red and yellow boxes in the dot plot (Box Y, Box Z; Figure 1C) show lack of sequence similarity between single amino acids near the beginning and middle of coding exon two. *Ilp2* in *D. simulans* has one protein-coding isoform (Ilp2-PA; Figure 1B). Isoform (Ilp2-PA) contains two protein-coding exons. Relative to the ortholog in *D. melanogaster* , the coding-exon number is conserved. The sequence of Ilp2-PA in *D. simulans* has 97.81% identity (E-value: 5e-97) with the protein-coding isoform Ilp2-PA in *D. melanogaster* , as determined by *blastp* (Figure 1C). Coordinates of this curated gene model are stored by NCBI at GenBank/BankIt (accession BK059551). These data are also archived in the CaltechDATA repository (see “Extended Data” section below).

## Extended Data

Description: FASTA, PEP, GFF. Resource Type: Model. DOI: 10.22002/qs6vt-d5d09
